# Recent Evolution of Coastal Tidal Flats and the Impacts of Intensified Human Activities in the Modern Radial Sand Ridges, East China

**DOI:** 10.3390/ijerph17093191

**Published:** 2020-05-04

**Authors:** Yifei Zhao, Qing Liu, Runqiu Huang, Haichen Pan, Min Xu

**Affiliations:** School of Marine Science and Engineering, Nanjing Normal University, Nanjing 210023, China; liuq@njnu.edu.cn (Q.L.); ruin_@sohu.com (R.H.); p1349609918@163.com (H.P.)

**Keywords:** tidal flats, coastal revolution, human activities, modern radial sand ridges

## Abstract

The coastal tidal flats of the modern Radial Sand Ridges (RSRs) are typical silt-muddy tidal flats in Central Jiangsu Province. These tidal flats play a critical role in coastline protection and biodiversity conservation, and against storm surges, but have recently been displaying drastic changes in geomorphic dynamics because of human activities. However, a comprehensive understanding of spatiotemporal changes in tidal flats in RSRs remains lacking. Hence, we employed a novel remote sensing method by obtaining the instantaneous high/low tide line positions from over 112 scenes of Landsat satellite images of the study area from 1975 to 2017, which were used to track the recent evolution of the coastal tidal flats in the modern RSRs over the past four decades. We found that the shoreline of the tidal flats showed an advanced seaward trend, and the waterline of the tidal flat presented a gradual process during different periods. The total tidal flat area in the study area showed an obviously decreasing trend overall, and approximately 992 km^2^ of the tidal flat was lost. We also found that the coastal tidal flats in the modern RSRs were generally undergoing erosion in the low tidal flats, especially in the Northern Swing and Southern Swing areas, while the high tidal flats showed a slowed accretionary change. Land reclamation was the main factor affecting the reduction in the tidal flat area, as the reclamation area has increased by 1300 km^2^, with an average of 35.14 km^2^/year. In addition, the erosion of the tidal flats was associated with a reduced sediment supply. Our findings will provide useful information for local managers and researchers to support future environmental management because increasing demand for land and rising sea levels are expected in the future.

## 1. Introduction

Coastal zones are an important interface for the interaction between land and sea, and are also the most rapid and sensitive zones in response to global changes and various land and sea dynamics [1]. Tidal flats, which are areas between the mean low tide and mean high tide [2,3], serve as the most important coastal geomorphic systems in the coastal zone and are widely distributed in many coastal zones across the world, including the coast of England [4], the Wadden coast in Northern Europe [5], the Amazon River mud bank in French Guiana [6], the Gomso Bay in Korea [7], and the Chinese coast [2,8,9]. They play an irreplaceable role in coastline protection and biodiversity conservation, and against storm surges and other natural disasters [10,11,12,13]. Meanwhile, tidal flats provide habitats for migrating birds and are reserved land resources for urban sprawl with exploding populations [14]. Because of this, tidal flats have received considerable attention from scientists in many parts of the world [9,11,13,15,16,17,18]. However, tidal flats are among the most highly vulnerable areas on Earth and are particularly sensitive to global changes [19]. Recently, coastal tidal flats have experienced drastic changes because of intensified natural factors, such as sea-level rise, and human activities, such as coastal reclamation, reduction in river sediment and subsidence and compaction of coastal land, which has led to land losses of more than 50% and as much as 80% of their original area in many regions of the world’s coastal tidal wetlands [20]. Additionally, a 20%–45% loss of salt marsh is predicted for the current century [21]. Hence, it is necessary and urgent to understand and predict the morphological evolution of tidal flats, and the response to increasing anthropogenic activities and natural factors.

The coastal tidal flats of modern Radial Sand Ridges are one of the largest silt-muddy coastal tidal flats in China and are distributed between the Sheyang River estuary and the Haozhi port in the Jiangsu Province [9,22]. In the past, the Yellow River entered the Southern Yellow Sea between 1128 and 1855 AD, providing abundant sediment for the construction of sand ridges [8,23], and the tidal flats of Jiangsu Province have advanced seaward, providing a favorable habitat for many migratory birds and benthos. However, the tidal flats of the modern Radial Sand Ridges have been affected by various factors over the past hundred years, which mainly include the following: (1) Change in sediment supply conditions caused by fluvial changes. The enormous amount of sediment supply has been lost in the study area due to the Yellow River returning to the Bohai Sea in 1855 [24]. In addition, the sediment load was also dramatically decreased at the Datong station of the Changjiang River after the completion of the Three Gorges Dam [25,26,27,28]. (2) The tidal flats of Radial Sand Ridges were reclaimed for farming, fisheries, salt production, wind plants, and harbor construction [29]. (3) Relative sea-level rise caused by global warming and land-based development (groundwater extraction and urban construction). The geomorphologically dynamic conditions of tidal flats in the Radial Sand Ridges have obviously shifted because of the combination of these factors, and the system state is changing from deposition to erosion [22]. Therefore, accurately evaluating the spatial distribution and geomorphological evolution of tidal flats is essential for the preservation and sustainability of tidal flat resources. However, tidal flats are usually spatially complex and temporally dynamic environments that are alternately submerged and exposed to air due to variations in tides, waves and sediment transport, and it is extremely difficult to obtain sufficient data through conventional field survey methods in a changing environment. Consequently, it is impossible to carry out continuous and comprehensive assessments of tidal flats on a long time scale.

Remote sensing techniques provide a near-continuous monitoring of shorelines in tidal flats, which is commonly applied on many global coasts [11,12,30,31]. The most representative methods are based on airborne LiDAR and InSAR images [32,33,34]. LiDAR can achieve relatively high vertical and planimetric accuracies, and InSAR is monitored across the full tidal range. However, terrain-based approaches are not applicable to large-area tidal flat mapping due to the scarcity of coastal DEMs with high resolution in spatial and temporal dominance, the spatial variation of water levels, rapidly changing tidal flat topography, and the high cost of airborne acquisitions. Some recent studies used the waterline method for generating topographic maps in the intertidal zone, which is currently considered to be the most useful approach. Chen et al. [35] manually selected the images with the highest or lowest shoreline to map the tidal flats of the Yangtze Estuary. Murray et al. [36] mapped the tidal flats around the world using a machine learning classifier mainly based on features of statistics of the Landsat-derived water indices on each individual pixel location. In addition, the waterline method has been widely applied in many regions throughout the world, including the Holderness coast in England, the Guiana coast in France, the Wadden coast in northern Europe, and the Yangtze estuary in China [37,38,39,40]. Thus, many methods have been developed to establish the location of the waterline and to map and monitor the status of tidal flats. It includes the following aspects: (1) a “same tide level comparison method” that is employed by using low tide images [41], which mainly compares the horizontal displacement of waterlines at low tide to study the trends in tidal flat evolution, (2) the waterline detection method (WDM), which involves constructing a digital elevation model by using a time series of satellite images [4,7,42,43], and (3) the tidal level correction method, which involves correction of the waterline to the multiyear mean high/low tide line [44,45]. However, these methods simplify the spatial variations in tidal level by using a single tidal height value for a whole scene, which will produce a significant error on a large scale since the tidal conditions differ in almost all the images. Some recent studies have attempted to overcome this dependence on tidal information. For example, the random forest classification algorithm and quantile synthesis method were used to classify tidal flats. However, there are significant differences in tidal flat area calculated by different algorithms [36,46]. Accurate evaluation of the change in tidal flats still faces great challenges on the regional scale. By combining the multiple satellite data from different phases, the accuracy of the obtained tidal flat extent was greatly improved, especially for studying the spatiotemporally dynamics of tidal flats by considering the tidal level spatial variations [11,14].

In the present study, we attempt to analyze the trends of the long-term morphological evolution of tidal flats in the Radial Sand Ridges over the period 1975–2017 based on a high-density time series of satellite images, which is a total of 112 multisource and multitemporal satellite images. Our specific objectives were (1) to study the long-term and continuous evolution of the tidal flat in the Radial Sand Ridges (RSRs) and (2) to discuss the influence of tidal flat reclamation activities and the change in sediment source. This study will aid in assessing the impacts of intensified human activities on coastal tidal flats in the modern Radial Sand Ridges, support the planning and operation of future environmental management, and provide a reference for sustainable tidal flat management.

## 2. Study Area

**Figure 1:**
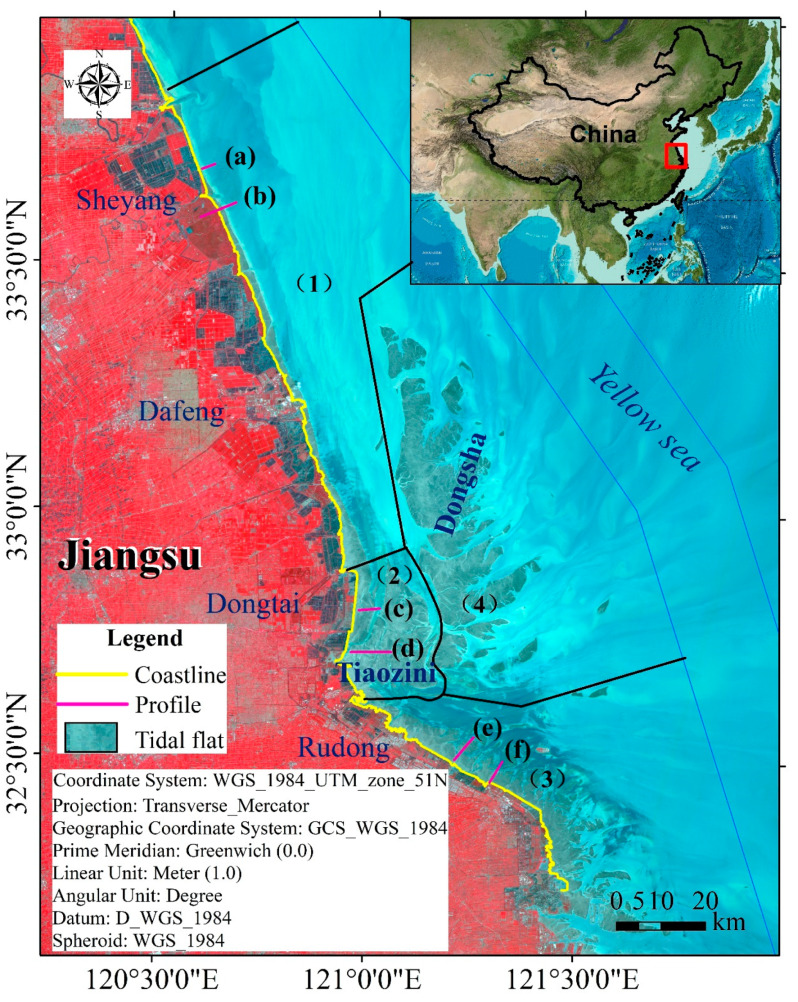
Geographical location map of the study area ((1) Northern Wing; (2) Inner part; (3) Southern Wing; (4) External part of the modern Radial Sand Ridges (RSRs)). (a), (b), (c), (d), (e) and (f) are typical cross sections in study area.

The modern Radial Sand Ridges (RSRs) (Figure 1), the largest tidal ridges on the Chinese continental shelf, are distributed off the Jiangsu coast in the western part of the South Yellow Sea, and the RSRs are situated from the Sheyang River estuary and extend south to the Haozhi Gang [9,47]. The RSRs consist of more than 70 sand ridges and tidal current channels with alternating grooves and ridges and range up to approximately 25 m in depth, covering a total area of approximately 22,470 km^2^ [47]. It is dominated by a progressive Poincaré wave from the East China Sea and an amphidromic system in the Yellow Sea, and all of the sand ridges are radially arranged, converging near Jianggang Harbor [48,49]. The study area is controlled by an irregular semidiurnal tidal wave, with the high tide lasting longer than the ebb tide and an average tidal range of 2.5–4.0 m. The tidal range is the widest off Jianggang–Yangkou Gang, reaching 6.45 m in the middle section, but gradually diminishing both northward and southward [9]. 

The coastal tidal flats of the RSRs are typical silt-muddy tidal flats, which are well-developed due to active tidal processes and abundant sediment supply [50,51]. Historically, the RSR field sediments were mostly derived from the old Yellow River and Yangtze River when the sediments were transported from the northern and southern sides of the system, respectively [3]. Consequently, the coastline of Jiangsu Province has advanced rapidly seaward. The Yellow River has been diverted to the Bohai Sea since 1855 AD, and the sediment load has decreased dramatically in the Yangtze River in recent decades, especially after the completion of the Three Gorges Dam [26,27,52]. Therefore, the geomorphology and sedimentary environments of the tidal flats and offshore sand ridges have dramatically changed and are undergoing a complex evolution in terms of scouring and siltation after losing a large sediment supply, such as the Yellow River and Yangtze River. Additionally, some areas of the Jiangsu coastal zone have been reclaimed.

## 3. Materials and Methods

### 3.1. Data Resources

**Figure 2:**
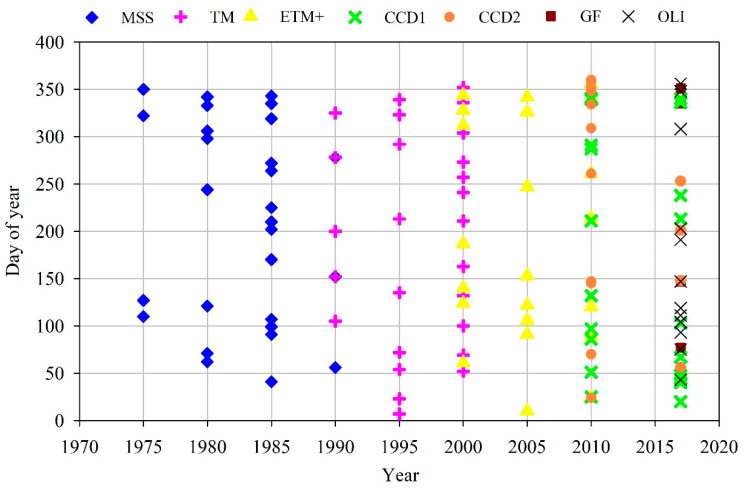
Acquisition dates of the satellite images used in this paper (Landsat multispectral scanner (MSS); Thematic Mapper (TM); Enhanced Thematic Mapper (ETM+); environment-1 and -2 satellite (CCD1, CCD2); High-resolution satellite imagery (GF) and Operational Land Imager (OLI)).

All available Landsat satellite images (including the Landsat Thematic Mapper (TM), Enhanced Thematic Mapper (ETM+), and Operational Land Imager (OLI_TIRS) images with a spatial resolution of 30 m and Landsat multispectral scanner (MSS) images with a spatial resolution of 80 m) from 1975 to 2017 were downloaded in this study (Figure 2). We selected the study area from the Sheyang River estuary in the north to the Haozhi Gang in the south, and one scene image basically covered the whole shoreline of the study area. A total of 112 multisource and multitemporal satellite images were analyzed, and all datasets were provided by the Earth Resources Observation and Science (EROS) center (http://glovis.usgs.gov/) and China Center for Resources Satellite Data and Application, CRESDA (http://www.cresda.com). Tidal action can cause tidal flats to become periodically submerged and exposed; therefore, we chose images carefully based on three conditions to obtain an accurate range of tidal flat resources: (1) all selected images were available within one year, except for those with cloudy coverage; (2) the spatial resolution of the image should be able to extract tidal beach resources over a small range and satisfy the study objectives on a larger spatial scale; and (3) the lowest available tidal levels were selected for different regions every year. The preprocessing of the satellite images (including image enhancement, haze deduction and geographic correction) was performed by using ERDAS IMAGING 9.2 software (Hexagon Geospatial, Madison, AL, USA). The detailed preprocessing procedures are presented in Zhang et al. [53].

### 3.2. Methods

**Figure 3:**
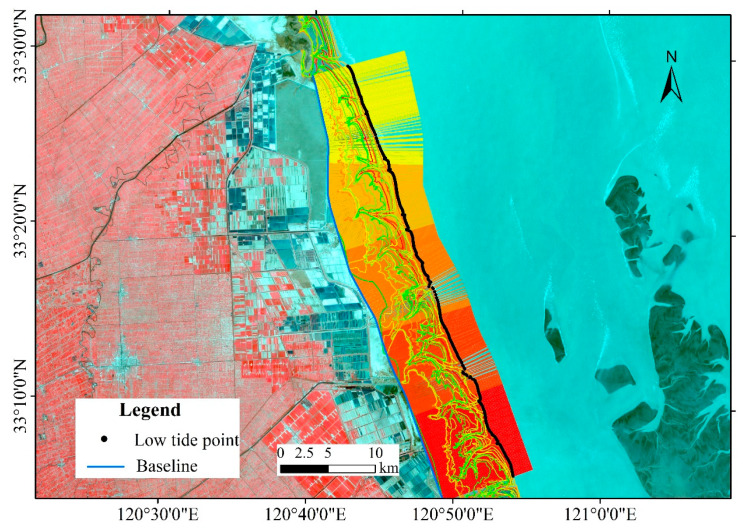
Determination of the tidal flat boundary.

**Figure 4:**
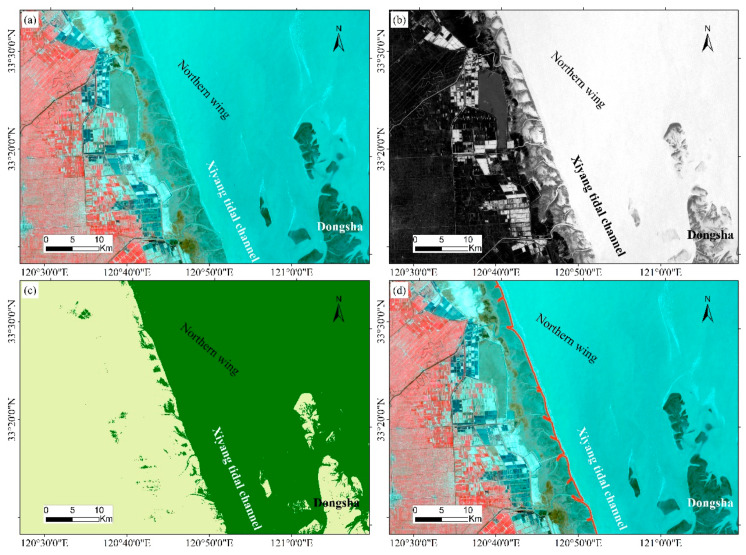
Extraction of the waterline location using normalized differential water body index (NDWI) threshold segmentation (**a**) original image; (**b**) the threshold segmentation result of NDWI; (**c**) NDWI binarization results; (**d**) the final waterline extraction results).

The instantaneous water boundary line of the remote sensing image is the instantaneous junction line of the sea and land under tidal fluctuation. Thus, it is necessary to obtain high and low tide lines to study the tidal flats. The high tidal line was typically determined based on some identifiable features, such as the wet-dry line, vegetation line, reclamation dikes, erosional scarps and the mean high water line [54,55,56]. Coastal tidal flats in the modern RSRs are accretionary tidal flats, where rare vegetation zones and heavily human reclaimed coasts are mainly distributed. Therefore, we chose the vegetation line and reclamation dike to be used as the high tide waterline (coastline) in our study. The extraction of the low tide waterline is more difficult due to the frequent occurrence of fluctuations in the satellite images [57]. Instantaneous lower tidal level images were used for every year to interpret low tide waterlines, e.g., first, the remote sensing images of different tidal levels were extracted for every year; second, a vertical line was set every 100 m using Digital Shoreline Analysis System (DSAS) software [8,58]; third, the Jenks Natural Breaks classification method was used to calculate the points on each vertical line and connect all the low tide points [59] (Figure 3). The method of combining visual interpretation and threshold segmentation was adopted to extract the waterline. Visual interpretation can ensure the accuracy and reliability of waterline extraction by extracting target objects from images through direct observations [60]. The threshold segmentation method selects the appropriate characteristic parameters and sets different thresholds to divide the remote sensing images into several categories of target regions and background regions with different gray levels according to the reflection characteristics of the different ground objects. Characteristic parameter selection and threshold setting are the key techniques in threshold segmentation. The common methods of waterline extraction include near infrared band density segmentation, the normalized differential vegetation index (NDVI), the normalized differential water body index (NDWI), and the new water body index (NWI) [61,62,63,64]. In this study, the NDWI proposed by Mcfeeters was used to extract the waterlines, which are dimensionless parameters comprising the linear combination of the near-infrared band and the green light band [62]. This method can effectively suppress the water impurity information and extract the boundary between the water body and nonwater body area. At present, it has been successfully applied in the waterline extraction of a high turbidity coastal zone [8,63]. The specific formula of NDWI is as follows:

(1) $$NDWI=\frac{\left(Green\;-\;NIR\right)}{\left(Green+NIR\right)}$$

where *Green* and *NIR* represent the green light band and the near infrared band, respectively. The threshold value was selected for binarization processing to extract the waterline by calculating the NDWI image. The optimal threshold value of the image was between −0.3 and −0.15. A threshold value of less than −0.3 could not distinguish the water body well, and an extraction result of greater than −0.15 was too fragmented. A visual interpretation was then used to verify and modify the automatically extracted waterline to ensure the accuracy of the waterline. The specific process is shown in the Figure 4.

**Figure 5:**
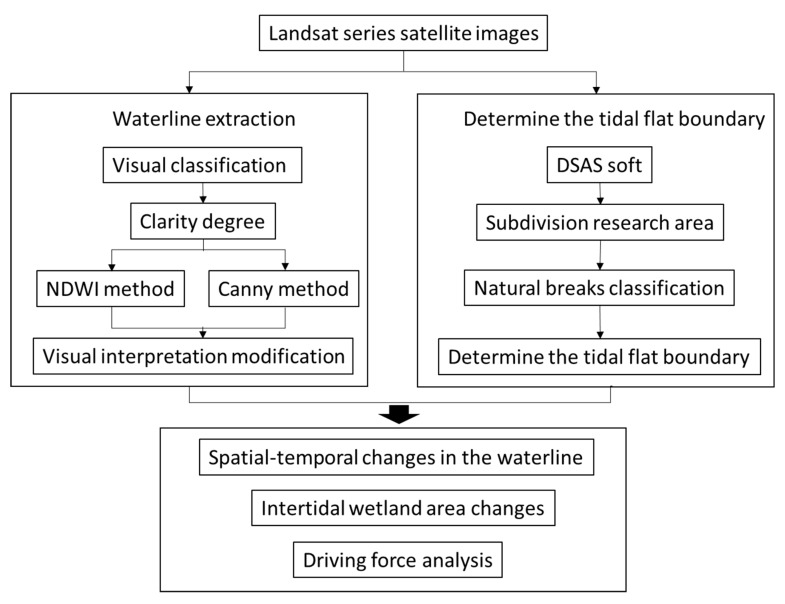
Data processing and analysis flow chart. DSAS: Digital Shoreline Analysis System.

Finally, the tidal flat data of a specific region were obtained for one year. Simultaneously, the reclamation data of tidal flats were manually extracted from Landsat images during different periods, and the flow chart of data processing and analysis is shown in Figure 5. Based on the morphologic characteristics of RSRs in the Jiangsu coastal area, we divided the study area into four parts: (1) the Northern Wing; (2) the Inner part; (3) the Southern Wing; and (4) the External part of the modern RSRs (Figure 1). 

### 3.3. Accuracy Assessment

We used the confusion matrix to assess the accuracy of tidal flat retrieval results, which is one of the most widely used accuracy evaluation methods in remote sensing and can simply summarize the main classification accuracy information [64]. The confusion matrix is a matrix of n rows and n columns, where each column represents the categories on the classification graph, and the total number of columns represents the number of data predicted for that category. Each row represents the category on the reference graph, the true category to which the data belong, and the total number of rows represents the number of data instances for that category. For a detailed explanation, the reader can refer to Powers and Zhao [65,66].

To ensure that the proportion of tidal flats and other features is appropriate, and ensure that all the real tidal flats are included, first, a 5-km buffer zone was made with the extracted tidal flats as the center, which could be used as the area for evaluating the accuracy of the extracted tidal flats. Afterwards, using the simple random sampling method, 240 random sampling points were generated by using the Create Random Points tool under the Data Management tool set in Arc GIS software (ESRI, Redlands, CA, USA) [67]. Reference data were derived by an independent analyst who labeled each sample point with tidal flats or other classes, based on the assessment of all available Landsat bands of the low-tide images and other available information (such as Google Earth) [68].

## 4. Results

### 4.1. Spatial-Temporal Changes in the Shoreline and Waterline

**Figure 6:**
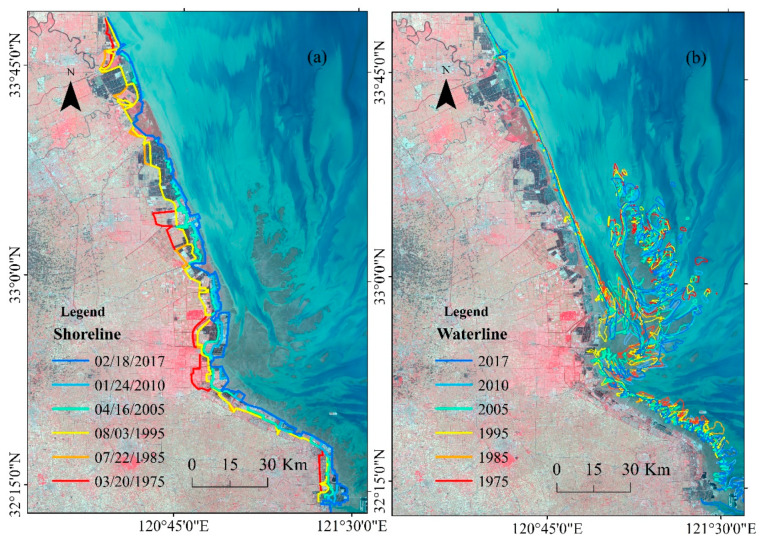
The changes in the shoreline and waterline during 1975–2017.

**Figure 7:**
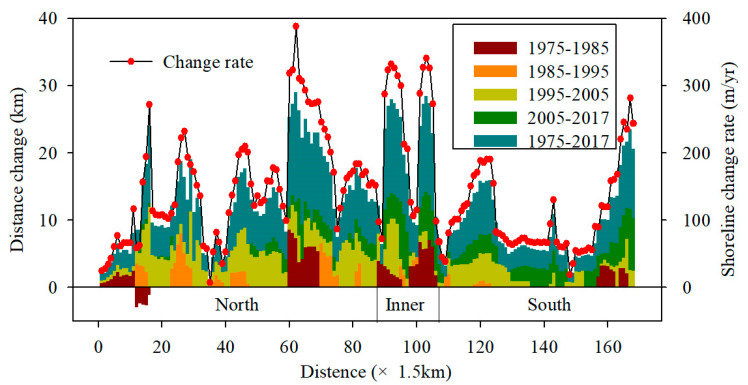
Spatial changes in the shoreline of the coastal tidal flats in the modern RSRs.

In general, the shoreline of the tidal flat in the modern radial sand ridges shows an overall advance toward the sea since 1975. Figure 6a and Figure 7 show that the shoreline has a trend of slow seaward sedimentation, and the sedimentation areas were mainly concentrated in the Sheyang estuary and Wanggang estuary in the Northern Wing between 1975 and 1995, with an average seaward advance of 91.15 m/year. In addition, the Tiaozini coast in the Inner part advanced seaward by 184.2 m/year, while the Southern Wing area of the tidal flat in the RSRs showed little change during this period. With the acceleration of coastal reclamation in Jiangsu Province during 1995–2005, the shoreline sedimentation rapidly accelerated, with an average seaward deposition of 2787 m and a sedimentation rate of 278.7 m/year; the most obvious increase occurred in the Northern Wing area, and the most rapid growth occurred from the Doulong port to Dongtai River estuary, with an average siltation rate of 357.1 m/year, followed by the Inner part, with an average siltation rate of 269.8 m/year. The Southern Wing had the slowest growth, and the average siltation rate was 171.5 m/year. In 2005–2017, the shoreline was still advancing seaward compared to that from 1995 to 2005, with a siltation rate of 149 m/year, but it showed a greater weakening trend. The siltation in the Northern Wing area was mainly concentrated in the southern part of Doulong Port, with an average siltation rate of 909.5 m/year; the siltation was the most obvious in the Inner part area during this period, which was dominated by the Tiaozini coast between the Liangduo River estuary and Fangtang River estuary, with an average rate of 340 m/year seaward and a maximum propulsion distance of 717 m/year from land to the sea. In the Southern Wing area, the coastline is advancing seaward with an obvious velocity, and the siltation rate is 196.3 m/year. In general, the coastline advanced seaward by an average of 6065 m between 1975 and 2017, with a siltation rate of 144.4 m/year, and the total shoreline change rate increased from 90.6 m/year during 1975–1985 to 149 m/year during 2005–2017. The average speed of seaward advancement shows a trend of “slow–fast–slow” trend.

In the 40–50 years since 1975, the waterline of the tidal flats in the RSRs generally presents a complex change characteristic (Figure 6b). This change is a gradual process in the remote sensing images during different periods. The waterline in the Northern Wing area has an overall retreating trend, with an overall retreat of approximately 2 km and an average annual retreat of approximately 47.6 m/year. Among them, the trend of retreat from north to south first decreased and then increased. The low tide line from the Sheyang estuary to the Xinyang Port has retreated by approximately 2 km, and the retreat of the scour from Xinyang Port toward the south has gradually declined, basically stabilizing near Doulong Port. The retreat of the waterline gradually increased from Wanggang to the Liangduo estuary, and the maximum recession distance reached approximately 3 km. The waterline in the Inner part of the area underwent a complex dynamic change during 1975–2017, and there was an erosional trend to the south in the north, but showed a trend of seaward advance in general, approximately 2–18 km seaward. The waterline of the Southern Wing in the RSRs shows fluctuation change characteristics, and there is a slight landward retreat trend. The waterline in the external sand ridge area shows a trend of retreat landward, and the erosional retreat is obvious in the west and northwest, with an average retreat rate of approximately 3–5 km. The alternation of erosion and siltation in the eastern and southeastern parts indicates that the areas are affected by the complex external dynamic environment. Consequently, the waterline of the tidal flats in the RSRs has presented complex variation characteristics in different regions since 1975. The distribution of erosion and siltation changes along the coast corresponds to the location characteristics of the north tidal flat near the erosive coast of the old Yellow River delta, the middle tidal flat near the inner margin area of the RSRs, and the south tidal flat near the Yangtze River, which is where the material supply decreases. That is, the Northern and Southern Wings retreated landward, the Inner part area advanced seaward, and the external sand ridge area shows alternate changes in erosion and deposition in terms of complex dynamic changes.

### 4.2. Intertidal Wetland Area Changes During 1980–2017

**Figure 8:**
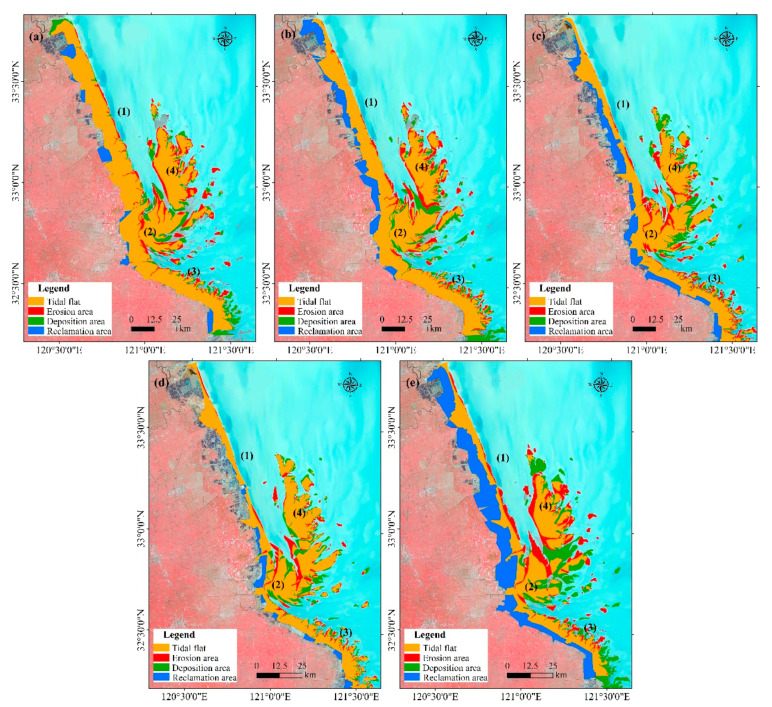
Spatial dynamics of the tidal flat in the modern RSRs ((1) Northern Wing; (2) Inner part; (3) Southern Wing; (4) External part of the modern RSRs). (**a**) 1980–1990; (**b**) 1990–2000; (**c**) 2000–2010; (**d**) 2010–2017; (**e**) 1980–2017.

The changes in tidal wetlands in the modern RSRs during four different periods are displayed in Table 1 and Figure 8. In general, the total area of the tidal flats showed an overall obviously decreasing trend in the study area, which declined by 992 km^2^ from 1980 to 2017. The maximal tidal flat area was 3338 km^2^ in 1980 and then started to notably decrease rapidly in 2000. In these periods, approximately 29.7% of the wetland area retreated, and the decrease rates were 26.8 km^2^/year. However, the area change in the tidal wetland presented obvious differences among the four subregions. 

The Northern Wing is an area that experienced a slight tidal flat decrease during 1980–1990 and has decreased sharply since 2000. Overall, the tidal flat area showed a significant decreasing trend, with a maximum area of 1175 km^2^ in 1980 and a minimum of 383 km^2^ in 2017, accounting for a 67.4% reduction. Moreover, erosion occurred in the Northern Wing region from the Sheyang estuary to Xinyang Port and south of Doulong Port between 1980 and 2017, and slight deposition occurred between Xinyang Port and Doulong Port. 

The Inner part of the tidal flat wetland is relatively stable with some fluctuations and it shows a trend of erosion in the north and deposition in the south during 1980–2017, with a maximum of 909 km^2^ in 2000 and a minimum of 842 km^2^ in 2017 (Table 1). Moreover, erosion mainly occurred in the northeast and south between 1980 and 1990, with an erosion area of 156.43 km^2^, and deposition occurred in the northwest and southeast, with a deposition area of 152.67 km^2^. Since 1990, the northern part of the inner fringe area has been continuously eroding with an erosion area of 634.88 km^2^, and deposition occurred in the east and south with a deposition area of 624.51 km^2^. 

The tidal flat area in the Southern Wing region showed an increasing trend from 1980 to 2000 and then decreasing after 2000. A maximum tidal flat area of 723 km^2^ occurred in 2000, a minimum of 537 km^2^ occurred in 2017, and the tidal area decreased by 25.7% from 2000–2017. Spatially, the dynamics of the tidal flat presented a complicated change, which is mainly manifested in the frequent change in erosion and deposition. In general, the tidal flat in the south wing region was dominated by deposition, with a mean depositional area of 152.43 km^2^ and a mean erosional area of 75.92 km^2^ from 1980–2000. During 2000–2017, the tidal flat was dominated by erosion, with an average depositional area of 92.34 km^2^ and an erosion area of 185.2 km^2^, which was approximately two times as large as the depositional area. 

The area change of tidal flats in the external sand ridge region of the modern RSRs can be divided into two periods, the decrease was obvious in the period 1980–2000, and indicated a slight growth after 2000. The tidal flat area decreased from 685 km^2^ in 1980 to a minimum of 515 km^2^ in 2000, falling by 24.8% compared with 1980. However, the area shows a slight increase of 11.8% from 2000 to 2007. Spatially, the dynamics of tidal flats also presented a complicated change, where erosion occurred in the western part of the outer fringe region and erosion/deposition occurred simultaneously in the eastern part during 1980–2007. In addition, the tidal flat shows an overall trend of moving from northwest to southeast. 

### 4.3. Evolution of the Typical Cross Sections in the Tidal Flats

**Figure 9:**
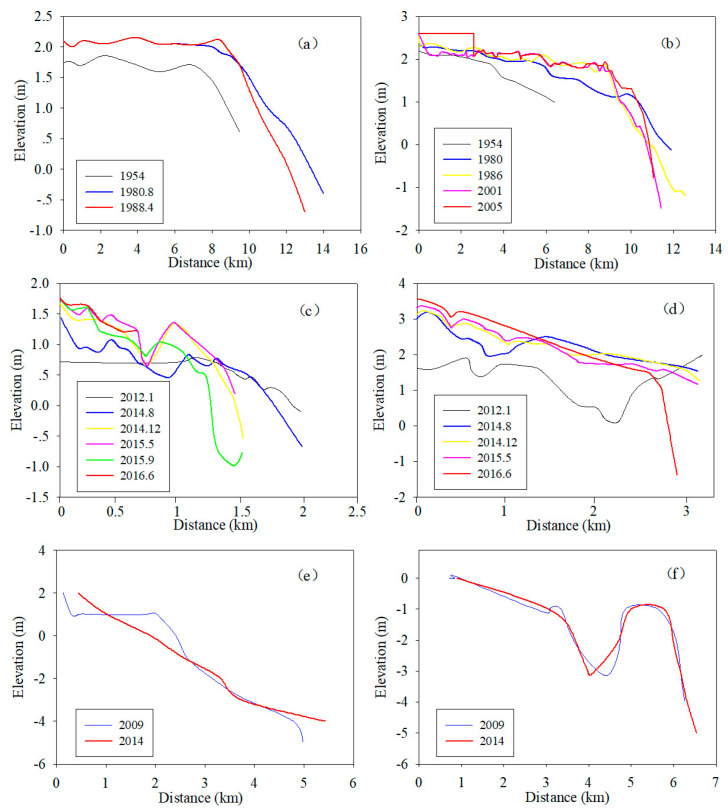
Change in the tidal flat profiles of the study area: (**a**) revised from Fang et al., 1992; (**b**) revised from Wang et al., 2003; (**c**,**d**) measurements in this study; (**e**,**f**) revised from Gu, 2018).

To further study the dynamic evolution of the tidal flats in the RSRs, typical section measurements of the tidal flats during different periods were compared and analyzed. Figure 9a shows the elevation change in the section in the northern part of the Xinyang Port from 1954–1988. In 1954–1980, the section of the tidal flat was in an obvious sedimentation state, with the sedimentation thickness of the tidal flat being approximately 30 cm overall, and the low tide line advanced seaward for approximately 1 km. However, there was almost no change in the tidal flats, and only localized siltation occurred near the high tide line from 1980 to 1988. The parts below the high tide line had been scoured, among which subtidal erosion was the most obvious. Figure 9b shows the elevation changes in the inner section of the Yancheng Nature Reserve on the south side of Xinyang Port from 1954 to 2005. The tidal flat shows an overall rapid continued deposition during 1954–1980, and the thickness and intensity of the sedimentation area were significantly greater than those in the northern part. From 1980 to 1986, the siltation of the tidal flat in the marsh grass and reed was relatively stable, the siltation of the tidal flat in the salt artemisia was slow, and the siltation of the tidal flat in the *Spartina alterniflora* Loisel was fast. However, scour was observed below the mean high water level and is most obvious near the low tide line. Since 1986, the side of the *Spartina alterniflora* Loisel is basically in a stable state along the shore, and the tidal flat near the high tide line shows further silting and advancement seaward because of the planting and expansion of *S. alterniflora*. The scouring and recession near the low tide line is obvious in the most recent 20 years, and the scouring recession range is approximately 800 m, with an average recession of 40 m/year. Consequently, in the past few decades, the siltation rate of the high tidal flats has decreased, erosion has occurred and the slope gradually becomes steepered in the low tidal flats of the Northern Wing area. In addition, the distance between the high tide line and the low tide line has been shortened, and the tidal area shows a decreasing trend. Figure 9c,d show the elevation changes of the Tiaozini section in the Inner part area of the RSRs from 2012 to 2014. The northern section of the tidal flat (Figure 8c) is undergoing continuous accretion in the 600 m range and shows scouring and siltation fluctuations from 600 m to the sea, which may be affected by tidal creek fluctuations. The southern section (Figure 9d) of the tidal flat shows obvious siltation within a range of 3000 m, while the scouring phenomenon occurred in the sea at 3000m. In general, the tidal flat of the Inner part area shows obvious siltation along the nearshore area, and the offshore area shows a state of scouring and siltation fluctuations that are affected by the tidal creeks. Moreover, the northern tidal flat in the offshore part is constantly scouring. Figure 8e,f show the elevation changes in the Southern Wing area of the RSRs from 2009 to 2014. The northern section (Figure 9e) shows the siltation nearshore (within 1000 m), scouring from 1000 to 2600 m and relatively stable from 2600 m and seaward. The southern section (Figure 9f) shows alternating changes in scouring and siltation, with small changes in the tidal flat surface and is relatively stable overall. As a result, the scouring and siltation changes in the south wing are complex, which is the dynamic variation of scouring and deposition, and the variation range is small.

## 5. Discussion

### 5.1. Influence of Sediment Source on the Tidal Flat

Several natural and anthropogenic factors have had obvious influences on the distribution and evolution of tidal flats. Changes in sediment load were identified as the major driving factors that influence tidal flats in China. The source of sediment material plays an important role in the scouring and siltation evolution of tidal flats the RSRs. In 1128, the Yellow River captured the Huai River into the sea from the northern part of Jiangsu Province, and the massive amount of sediment made the north coast of the northern coast advance rapidly seaward [69,70]. Since the shift of the Yellow River into the Bohai Sea in 1855 due to natural breaching [8,71,72], the sediment source of the Yellow River into the south Yellow Sea was greatly reduced and the local energetic hydrodynamic conditions changed. The coast of Jiangsu Province entered an unprecedented adjustment stage, which resulted in the abandoned delta coast in Northern Jiangsu Province experiencing a severe marine erosion and shoreline retreat [24,73,74,75]. Yu et al. (1986) estimated the total volume of coastal erosion, including nearshore areas, at 4.4 × 10^10^ m^3^ from 1855 to 1962 [73]; Wang and Aubrey (1987) indicated that an inland shoreline retreat of approximately 17 km occurred between 1855 and the 1980s [24]. The erosional range from the protuberant coast of the abandoned Yellow delta gradually expanded to both sides, and the near coast of the Sheyang estuary became a transitional coast that was transformed from siltation to erosion [22]. The tidal flats show a depositional trend in the high tide zone and erosion in the low tide zone, which cause the tidal flat to steepen and narrow, and the tidal flat area was reduced. In addition, the influence of runoff and sediment load entering the sea was gradually weakened since the shift of the river into the Bohai Sea, and the tidal dynamics along the Jiangsu coast became the main driving force affecting the sedimentary environment of the region. The Xiyang tidal channel extended continuously to the south, resulting in the continuous erosion of the tidal front of the Northern Wing area in the RSRs. Moreover, only a small part of the sediment from the Yangtze River diffuses along with the runoff to the north during summer and autumn [75], but the transport distance is short and the contribution to the southern part of the Southern Wing area is small. However, the sediment load of the Yangtze River into the sea has significantly decreased since the 1950s. The sediment load decreased from 500 million tons in the 1950s to 124 million tons during the impoundment of the Three Gorges Dam in 2003 [26,76], which resulted in a further decline in the material supplied by the Yangtze River to the coastal tidal plain of Jiangsu Province. 

### 5.2. Influence of Reclamation Activity

**Figure 10:**
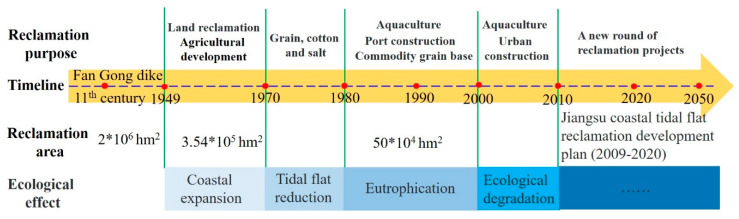
Reclamation history of the tidal flats in the Jiangsu Province.

Tidal flats are an important part of coastal wetlands in Jiangsu Province and are one of the most important natural resources. The tidal flat reclamation project is the main way to alleviate the contradiction between people and land in Jiangsu Province. Historically, the reclamation area of coastal tidal flats in Jiangsu Province reached 2 × 10^6^ hm^2^ [77]. Since 1949, large-scale reclamation in Jiangsu has occupied a large number of tidal flat resources and become an important factor affecting the change in tidal flat areas (Figure 10). During this period, the large-scale reclamation of tidal flats experienced several stages: (1) in the 1950–1960s, the major reclamation pattern was the construction of tidal dikes, the reclamation of wasteland and the development of agriculture; (2) in the 1970s, the agriculture, grain, cotton and salt industries were mainly being developed; (3) since the 1980s, the area was mainly been used for salt, grain and cotton, breeding and port construction; and (4) large-scale land reclamation has promoted the rapid development of the regional economy since the beginning of the 21st century, and the newly added land is mainly used for industrialization and port construction. According to the reclamation development plan for coastal tidal flats in Jiangsu Province, the goal of reclamation within a larger space will be realized. According to the reclamation situation of the Northern Jiangsu Province coast in the RSRs since 1980, the total reclamation area shows an increasing trend, especially the rapid increase from 1980 to 2010 and the slowing down from 2010 to 2017. Over the past 37 years, the reclamation area of the RSR coast has increased by 1300 km^2^, with an average annual increase of 35.14 km^2^. The reclamation area is distributed along all the coastline sections of the sand ridges, and the reclamation area of the Northern Wing area and the Inner part area is larger than that of the Southern Wing area. Specifically, the reclamation area of the study area was 179.51 km^2^ from 1980 to 1990, which was mainly distributed in the Northern Wing, the Inner part, and the Southern Wing, while the reclamation area of the Northern Wing was larger than that of the Inner part and the Southern Wing. The reclamation area of the study area was 424 km^2^ between 1990 and 2000, which was more than twice the area during the 1990s, and the reclamation area was mainly distributed along the whole coast of the Northern Wing area. From 2000 to 2010, the reclamation area reached a maximum value of nearly 37 years in the study area, and the whole coast of the RSRs underwent different degrees of reclamation, especially from Xinyang Port to Dongtai estuary in the Northern Wing area, with the accumulated newly increased reclamation area reaching 526 km^2^ during this period. During 2010–2017, the reclamation area showed a more significant decreasing trend than before 2010, with a cumulative newly increased reclamation area of 170.86 km^2^. The reclamation area was mainly concentrated in the Inner part of the coast of the RSRs, and a small amount of reclamation was mainly distributed in the south wing. In addition, from the reclamation distribution map of the RSRs, the reclamation activities on the inner side of the natural coastline of the Northern Wing area have approached the coastline. Therefore, the high-intensity reclamation and development of the tidal flats has resulted in the loss of high-tide flat and occupied the tidal flat resources of the study area. Reclamation and development have also resulted in fundamental changes in the tidal flat pattern and a further reduction in the tidal flat area.

### 5.3. Influence of other Factors

The change in sedimentary dynamics is an important factor in the evolution of coastal tidal flats [75]. The spatial and temporal scales of the RSRs have been greatly adjusted since 1855, the influence of the runoff and sediment load into the sea on the coast has gradually weakened, and the leading role of the offshore tidal current has gradually strengthened. The Xiyang water channel is a shoreland tidal water channel in the Northern Wing area of the RSRs, which extends continuously to the south, causing the low tidal flat to be eroded under the guidance of the nearshore hydrodynamic force, and the eroded materials are transported southward under the action of the coastal current in Jiangsu Province [22]. The Southern Wing area of the RSRs is adjacent to the Xiaomiaohong water channel, which is a coastal tidal waterway in the south wing area. In recent decades, it has been moving to the shore continuously seaward, which has damaged the balance of the tidal flat profile and caused bank slope erosion. Moreover, the Inner part area of the RSRs belongs to the strong tidal current coast, which results in the erosion of the tidal flats when strong currents strike the concave bank of the tidal flats.

Global warming will accelerate the rise in sea level and increase the intensity and frequency of storm surges and waves, which will lead to greater intensity and a probability of coastal erosion. The average sea-level rise rate in the coastal areas of Jiangsu is 2.2 mm/year, which is one of the most obvious areas of relative sea level rise in China [78]. This rise will lead to a reduction in the sea dike defense ability in the coastal areas of Jiangsu, the expansion of coastal erosion areas, and the loss of coastal tidal flat resources.

A remote sensing method was used for generating topographic maps of the tidal flat by the waterline method in this study. Some limitations that should be addressed. However, these uncertainties in the results seem to be inevitable. First, this work was limited by the quality and quantity of the Landsat images, which cannot resolve the rapidity of tidal variations. For example, the 16-day revisit period of Landsat images includes cloudy coverage images. Second, the low tide boundary line obtained at the low tide moments was used to determine whether the tidal flat range is more accidental. Due to the influence of weather, terrain, and other factors, the tidal movement in different regions at the same time is different. With the increasing number of satellite image sources, including Landsat 8, Sentinel-1 and 2, and Worldview satellite images in the future, more high-quality satellite images are available, the temporal resolution of tidal flats is improved, and the potential for use in other coastal areas around the world is improved. Furthermore, the final dataset of the tidal flat that was obtained in this study has the potential to be applied widely (1) to estimate tidal topographic changes, (2) to investigate the mechanisms of water and sediment transport and exchange, and (3) to support morphological evolution models in coastal areas.

## 6. Conclusions

It is crucial to understand the evolutionary trends of tidal flat development for local ecological protection, environmental management, and regional sustainable development. In this study, we present the detailed spatiotemporal dynamics of the geomorphological evolution of coastal tidal flats in the modern RSRs based on a novel remote sensing method by obtaining the instantaneous waterline positions from over 112 scenes of Landsat satellite images of the study area from 1975 to 2017. Our results suggest that the shoreline of the tidal flat shows an overall advancement seaward over the last 42 years, whereas the tidal flat waterline presents a gradual process in remote sensing images during different periods. According to statistics, approximately 29.7% of the wetland area retreated in these periods, and the rate of decrease was 26.8 km^2^/year. In addition, the coastal tidal flats of modern RSRs are generally undergoing erosion in the low tidal flats, especially in the Northern and Southern Wings, while the high tidal flats are still in an accretionary phase, but the rate of deposition is slowing. The geomorphological evolution of the coastal tidal flat in the modern RSRs has mainly been controlled by land reclamation. The sediment supply, hydrodynamics, and sea-level rise have also had a significant effect. Ultimately, our study may contribute to providing comprehensive knowledge related to the evolution of tidal flats in the modern RSRs. The reduction in the tidal flat area will seriously damage the habitats of migrating birds and the ecosystem in this study. Therefore, it is necessary for better coastal protection and further environmental management in coastal tidal flats in the future. This study used the waterline method to extract the tidal flat range of the RSRs in Northern Jiangsu Province and will help assess the effect of well-known reclamation on Coastal Jiangsu Province, identify the mechanisms underlying the evolution of tidal flats, and support the planning and operation of future coastal development. In addition, we found that the waterline method has the advantage of enabling the generation of an intertidal range over large areas, relatively rapidly and inexpensively at a larger spatial scale, and the potential for use in other coastal areas around the world. With the number of satellite image sources, more high-quality satellite images will be available in the future, which can greatly improve the vertical accuracy and spatial resolution of tidal flats.

## Figures and Tables

**Table 1:** Area change at different periods during 1980–2017 in the study area (unit: km^2^).

Period	Reclamation Area	I Region		II Region		III Region		IV Region	
		Accretion	Erosion	Accretion	Erosion	Accretion	Erosion	Accretion	Erosion
1980–1990	179.51	62.00	47.91	152.67	156.43	129.42	59.68	104.92	165.33
1990–2000	424.00	23.00	398.00	227.19	170.66	175.43	92.16	93.38	203.65
2000–2010	526.00	21.00	306.69	194.98	254.32	77.25	249.40	160.49	126.71
2010–2017	170.86	30.39	69.40	202.34	209.90	107.43	120.97	119.05	84.18
1980–2017	1300.37	36.15	828.34	370.43	397.49	230.29	302.33	155.42	257.45

## References

[1] Wang Y., Zhu D.K., Zhou L.F., Wang X.Y., Jiang S.L., Li H.Y., Shi B.W., Zhang Y.Z. (1998). Sedimentary characteristics and evolution of radial sand ridges in the south yellow sea. Sci. China Ser. D.

[2] Chen J.Y., Cheng H.Q., Dai Z.J., Doeke E. (2008). Harmonious development of utilization and protection of tidal flats and wetlandsda case study in Shanghai area. China Ocean. Eng..

[3] Wang X., Xiao X., Zou Z., Chen B., Ma J., Dong J., Li X. (2018). Tracking annual changes of coastal tidal flats in China during 1986–2016 through analyses of Landsat images with Google Earth Engine. Remote Sens. Environ..

[4] Mason D.C., Davenport I.J., Robinson G.J., Flather R.A., McCartney B.S. (1995). Construction of an inter-tidal digital elevation model by the ‘Water-Line’Method. Geophys. Res. Lett..

[5] van Loon-Steensma J.M. (2015). Salt marshes to adapt the flood defences along the Dutch Wadden Sea coast. Mitig. Adapt. Strateg. Glob. Chang..

[6] Anthony E.J., Gardel A., Gratiot N., Proisy C., Allison M.A., Dolique F., Fromard F. (2010). The amazon-influenced muddy coast of South America: A review of mud-bank–shoreline interactions. Earth-Sci. Rev..

[7] Ryu J.H., Kim C.H., Lee Y.K., Won J.S., Chun S.S., Lee S. (2008). Detecting the intertidal morphologic change using satellite data. Estuar. Coast. Shelf Sci..

[8] Liu Y., Li M., Mao L., Cheng L., Chen K. (2013). Seasonal pattern of tidal-flat topography along the Jiangsu middle coast, China, using HJ-1 optical images. Wetlands.

[9] Xu M., Meng K., Zhao Y., Zhao L. (2018). Sedimentary Environment Evolution in East China’s Coastal Tidal Flats: The North Jiangsu Radial Sand Ridges. J. Coast. Res..

[10] Murray N.J., Clemens R.S., Phinn S.R., Possingham H.P., Fuller R.A. (2014). Tracking the rapid loss of tidal wetlands in the Yellow Sea. Front. Ecol. Environ..

[11] Li X., Zhang X., Qiu C., Duan Y., Liu S., Chen D., Zhang L., Zhu C. (2020). Rapid Loss of Tidal Flats in the Yangtze River Delta since 1974. Int. J. Environ. Res. Public Health.

[12] Zhao C., Qin C.-Z., Teng J. (2020). Mapping large-area tidal flats without the dependence on tidal elevations: A case study of Southern China. Isprs J. Photogramm. Remote Sens..

[13] Sagar S., Roberts D., Bala B., Lymburner L. (2017). Extracting the intertidal extent and topography of the Australian coastline from a 28 year time series of Landsat observations. Remote Sens. Environ..

[14] Zhang X., Zhang Y., Zhu L., Chi W., Yang Z., Wang B., Lu Z. (2018). Spatial-temporal evolution of the eastern Nanhui mudflat in the Changjiang (Yangtze River) Estuary under intensified human activities. Geomorphology.

[15] Tong S.S., Deroin J., Pham T.L. (2020). An optimal waterline approach for studying tidal flat morphological changes using remote sensing data: A case of the northern coast of Vietnam. Estuar. Coast. Shelf Sci..

[16] Dyer K.R., Christie M.C., Feates N., Fennessy M.J., Pejrup M., van der Lee W. (2000). An investigation into processes influencing the morphodynamics of an intertidal mudflat, the dollard estuary, The Netherlands: I. hydrodynamics and suspended sediment. Estuar. Coast. Shelf Sci..

[17] Kirwan M.L., Megonigal J.P. (2013). Tidal wetland stability in the face of human impacts and sea-level rise. Nature.

[18] Xie W., He Q., Zhang K., Guo L., Wang X., Shen J. (2018). Impacts of human modifications and natural variations on short-term morphological changes in estuarine tidal flats. Estuar. Coast..

[19] Spencer T., Schuerch M., Nicholls R.J., Hinkel J., Lincke D., Vafeidis A.T., Reef R., Mcfadden L., Brown S. (2016). Global coastal wetland change under sealevel rise and related stresses: The DIVA Wetland Change Model. Glob. Planet. Chang..

[20] Airoldi L., Beck M.W. (2007). Loss, status and trends for coastal marine habitats of Europe. Oceanogr. Mar. Biol..

[21] Craft C., Clough J., Ehman J., Joye S.B., Park R.A., Pennings S., Guo H., Machmuller M.B. (2009). Forecasting the effects of accelerated sea-level rise on tidal marsh ecosystem services. Front. Ecol. Environ..

[22] Xu M., Li P.Y., Lu P.D. (2012). Research on Appropriate Reclamation Scale of Prograding Tidal Flat: A Case Study of Jiangsu Province.

[23] Zhang R. (1984). Land-forming history of the Huanghe River delta and coastal plain of northern Jiangsu. Acta Geogr. Sin..

[24] Wang Y., Aubrey D.G. (1987). The characteristics of the China coastline. Cont. Shelf Res..

[25] Yang Z.S., Wang H.J., Saito Y., Milliman J.D., Xu K., Qiao S., Shi G. (2006). Dam impacts on the Changjiang (Yangtze) River sediment discharge to the sea: The past 55 years and after the Three Gorges Dam. Water Resour. Res..

[26] Zhao Y., Zou X., Gao J., Xu X., Wang C., Tang D., Wu X. (2015). Quantifying the anthropogenic and climatic contributions to changes in water discharge and sediment load into the sea: A case study of the Yangtze River, China. Sci. Total Environ..

[27] Guo L., Su N., Townend I., Wang Z.B., Zhu C., Wang X., He Q. (2019). From the headwater to the delta: A synthesis of the basin-scale sediment load regime in the Changjiang River. Earth-Sci. Rev..

[28] Peng T., Tian H., Singh V.P., Chen M., Liu J., Ma H., Wang J. (2020). Quantitative assessment of drivers of sediment load reduction in the Yangtze River basin, China. J. Hydrol..

[29] Chen J., Zhang C.K., Lin K., Ding X.R., Yuan R.H. (2011). Reclamation and development of coastal tidal flats in Jiangsu Province. J. Hohai Univ. Nat. Sci..

[30] Darwish K., Smith S.E., Torab M., Monsef H., Hussein O. (2016). Geomorphological changes along the Nile Delta coastline between 1945 and 2015 detected using satellite remote sensing and GIS. J. Coast. Res..

[31] Li W., Gong P. (2016). Continuous monitoring of coastline dynamics in western Florida with a 30-year time series of Landsat imagery. Remote Sens. Environ..

[32] Guo Q.H., Li W.K., Yu H., Alvarez O. (2010). Effects of topographic variability and Lidar sampling density on several DEM interpolation methods. Photogramm. Eng. Remote Sens..

[33] Deronde B., Houthuys R., Debruyn W., Fransaer D., Lancker V.V., Henriet J.P. (2006). Use of airborne hyperspectral data and laser scan data to study beach morphodynamics along the Belgian coast. J. Coast. Res..

[34] Slater J.A., Garvey G., Johnston C. (2006). The SRTM data “finishing” process and products. Photogramm. Eng. Remote Sens..

[35] Chen Y., Dong J.W., Xiao X.M., Zhang M., Tian B., Zhou Y.X., Li B., Ma Z.J. (2016). Land claim and loss of tidal flats in the Yangtze Estuary. Sci. Rep..

[36] Murray N.J., Phinn S.R., DeWitt M., Ferrari R., Johnston R., Lyons M.B., Clinton N., Thau D., Fuller R.A. (2019). The global distribution and trajectory of tidal flats. Nature.

[37] Mason D.C., Scott T.R., Dance S.L. (2010). Remote sensing of intertidal morphological change in Morecambe Bay, U.K., between 1991 and 2007. Estuarine. Coast. Shelf Sci..

[38] Anthony E.J., Dolique F., Gardel A., Gratiot N., Proisy C., Polidori L. (2008). Nearshore intertidal topography and topographic-forcing mechanisms of an Amazon-derived mud bank in French Guiana. Cont. Shelf Res..

[39] Heygster G., Dannenberg J., Notholt J. (2010). Topographic mapping of the German tidal flats analyzing SAR images with the waterline method. IEEE Trans. Geosci. Remote Sens..

[40] Zhao B., Guo H.Q., Yan Y., Wang Q., Li B. (2008). A simple waterline approach for tidelands using multi-temporal satellite images: A case study in the Yangtze Delta. Estuar. Coast. Shelf Sci..

[41] Wang X.D., Fang C., Kang H., Xie H.L., Liu F.T., Meng L. (2014). Remot sensing monitoring of the Caofeidian tidal zone evolution. Mar. Sci. Bull..

[42] Scott T.R., Mason D.C. (2007). Data assimilation for a coastal area morphodynamic model: Morecambe Bay. Coast. Eng..

[43] Kang Y., Ding X., Xu F., Zhang C., Ge X. (2017). Topographic mapping on large-scale tidal flats with an iterative approach on the waterline method. Estuar. Coast. Shelf Sci..

[44] Wang X.L., Zhang J., Chu J.L. (2005). Extraction of remotely sensed information of island intertidal zone and wetland. Adv. Mar. Sci..

[45] Fang C., Sun X.M., Kang H., Ge D.Q., Wang X.D., Xie H.L. (2015). Application of remote sensing technology to geological environment investigation in the Caofiedian coastal zone. Hydrogeol. Eng. Geol..

[46] Zhang K., Dong X., Liu Z., Gao W., Hu Z., Wu G. (2019). Mapping tidal flats with Landsat 8 images and google earth engine: A case study of the China’s eastern coastal zone circa 2015. Remote Sens..

[47] Wang Y. (2002). Radiative Sandy Ridge Field on Continental Shelf of the Yellow Sea.

[48] Xing F., Wang Y.P., Wang H.V. (2012). Tidal hydrodynamics and fine-grained sediment transport on the radial sand ridge system in the southern Yellow Sea. Mar. Geol..

[49] Xu F., Tao J., Zhou Z., Coco G., Zhang C. (2016). Mechanisms underlying the regional morphological differences between the northern and southern radial sand ridges along the Jiangsu Coast, China. Mar. Geol..

[50] Liu X.Y., Gao J.H., Bai F.L., Liu Z.Y., Pan S.M. (2008). Grain size information in different evolution periods of Xinyanggang tidal flat in Jiangsu province. Mar. Geol. Q. Geol..

[51] Wang Y., Zhu D.K. (1990). Tidal flats of China. Quat. sci..

[52] Yang S.L., Belkin I.M., Belkina A.I., Zhao Q.Y., Zhu J., Ding P.X. (2003). Delta response to decline in sediment supply from the yangtze river: Evidence of the recent four decades and expectations for the next half-century. Estuar. Coast. Shelf Sci..

[53] Zhang X.D., Zhang Y.X., Ji Y., Zhang Y.W., Yang Z.S. (2016). Shoreline change of the Northern Yellow River (Huanghe) Delta after the latest deltaic course shift in 1976 and its influence factors. J. Coast. Res..

[54] Chu Z.X., Sun X.G., Zhai S.K., Xu K.H. (2006). Changing pattern of accretion/erosion of the modem Yellow River (Huanghe) subaerial delta, China: Based on remote sensing images. Mar. Geol..

[55] Pajak M.J., Leatherman S. (2002). The high water line as shoreline indicator. J. Coast. Res..

[56] Liu Y.C., Zhang Y. (2010). Study on the tidal flat evolution through changes of coastline and beach line of Sheyang River estuary by the remote sensing. Mar. Sci. Bull..

[57] Zhang X.D., Yang Z.S., Zhang Y.X., Ji Y., Wang H.M., Lv K., Lu Z.Y. (2017). Spatial and temporal shoreline changes of the southern Yellow River (Huanghe) Delta in 1976–2016. Mar. Geol..

[58] Thieler E.R., Himmelstoss E.A., Zichichi J.L., Ergul A. The Digital Shoreline Analysis System (DSAS) version 4.0-an ArcGIS extension for calculating shoreline change (No. 2008-1278). https://cmgds.marine.usgs.gov/publications/DSAS/of2008-1278/.

[59] Yu B.H., Lv C.H., Lv T.T., Yang A.Q., Liu C. (2009). Regional differentiation of vegetation change in the Qinghai-Tibet Plateau. Prog. Geog..

[60] Niedermeier A., Hoja D., Lehner S. (2005). Topography and morphodynamics in the German Bight using SAR and optical remote sensing data. Ocean Dynam..

[61] Frouin R., Schwindling M., Deschamps P.Y. (1996). Spectral reflectance of sea foam in the visible and near-infrared: In situ measurements and remote sensing implications. J. Geophys. Res. Oceans.

[62] McFeeters S.K. (1996). The use of the Normalized Difference Water Index (NDWI) in the delineation of open water features. Int. J. Remote Sens..

[63] Wang Y., Hou X., Shi P., Yu L. (2013). Detecting shoreline changes in typical coastal wetlands of Bohai Rim in North China. Wetlands.

[64] Foody G.M. (2002). Status of land cover classification accuracy assessment. Remote Sens. Environ..

[65] Powers D.M. (2011). Evaluation: From precision, recall and F-measure to ROC, informedness, markedness and correlation. J. Mach. Learn. Technol..

[66] Zhao Y.S. (2003). Principles and Methods of Remote Sensing Application Analysis.

[67] Congalton R.G. (1991). A review of assessing the accuracy of classifications of remotely sensed data. Remote Sens. Environ..

[68] Murray N.J., Phinn S.R., Clemens R.S., Roelfsema C.M., Fuller R.A. (2012). Continental scale mapping of tidal flats across East Asia using the Landsat archive. Remote Sens..

[69] Zhang R.S. (1984). Relation between changes of submarine sand ridge field and development of coast near Jianggang, Jiangsu. J. Nanjing Univ. Nat. Sci..

[70] Xu J. (2003). Growth of the Yellow River Delta over the past 800 years, as influenced by human activities. Geogr. Ann. Ser. A Phys. Geogr..

[71] Ren M., Shi Y. (1986). Sediment disharge of the Yellow River (China) and its effect on the sedimentation of the Bohai and the Yellow Sea. Cont. Shelf Res..

[72] Saito Y., Wei H., Zhou Y., Nishimura A., Sato Y., Yokota S. (2000). Delta progradation and chenier formation in the Huanghe (Yellow River) delta, China. J. Asian Earth Sci..

[73] Yu Z., Lin L., Chen D. (1986). Erosion of Old Yellow River submerged delta in northern Jiangsu Province, China. Trans. Jpn. Geomorphol. Union..

[74] Saito Y., Yang Z.S. Historical change of the Huanghe (Yellow River) and its impact on the sediment budget of the East China Sea. Proceedings of the International Symposium on Global Fluxs of Carbon and its Related Substances in the Coastal Sea-Ocean Atmosphere System.

[75] Zhang R.S., Lu L.Y., Wang Y.H. (2002). The mechamism and trend of coastal erosion of Jiangsu Province in China. Geogr. Res..

[76] Luo X.X., Yang S.L., Zhang J. (2012). The impact of the Three Gorges Dam on the downstream distribution and texture of sediments along the middle and lower Yangtze River (Changjiang) and its estuary, and subsequent sediment dispersal in the East China Sea. Geomorphology..

[77] Agricultural Resources Development Bureau of Jiangsu Province (1999). Coastal Reclamation Area of Jiangsu Province.

[78] Wang J. (2012). Coastal Mudflat of Jiangsu Province and Its Utilization Potential.

